# Supplementary material for: Dendritic Cell Lineage Potential in Human Early Hematopoietic Progenitors
Source: Cell Rep. 2017 Jul 18;20(3):529–37. doi: 10.1016/j.celrep.2017.06.075 (PMC5529209; doi:10.1016/j.celrep.2017.06.075)
Supplement: Document S1. Supplemental Experimental Procedures and Figures S1–S4 [file mmc1.pdf]

Cell Reports, Volume *20*

Supplemental Information

Dendritic Cell Lineage Potential

in Human Early Hematopoietic Progenitors

Julie Helft, Fernando Anjos-Afonso, Annemarthe G. van der Veen, Probir Chakravarty, Dominique Bonnet, and Caetano Reis e Sousa

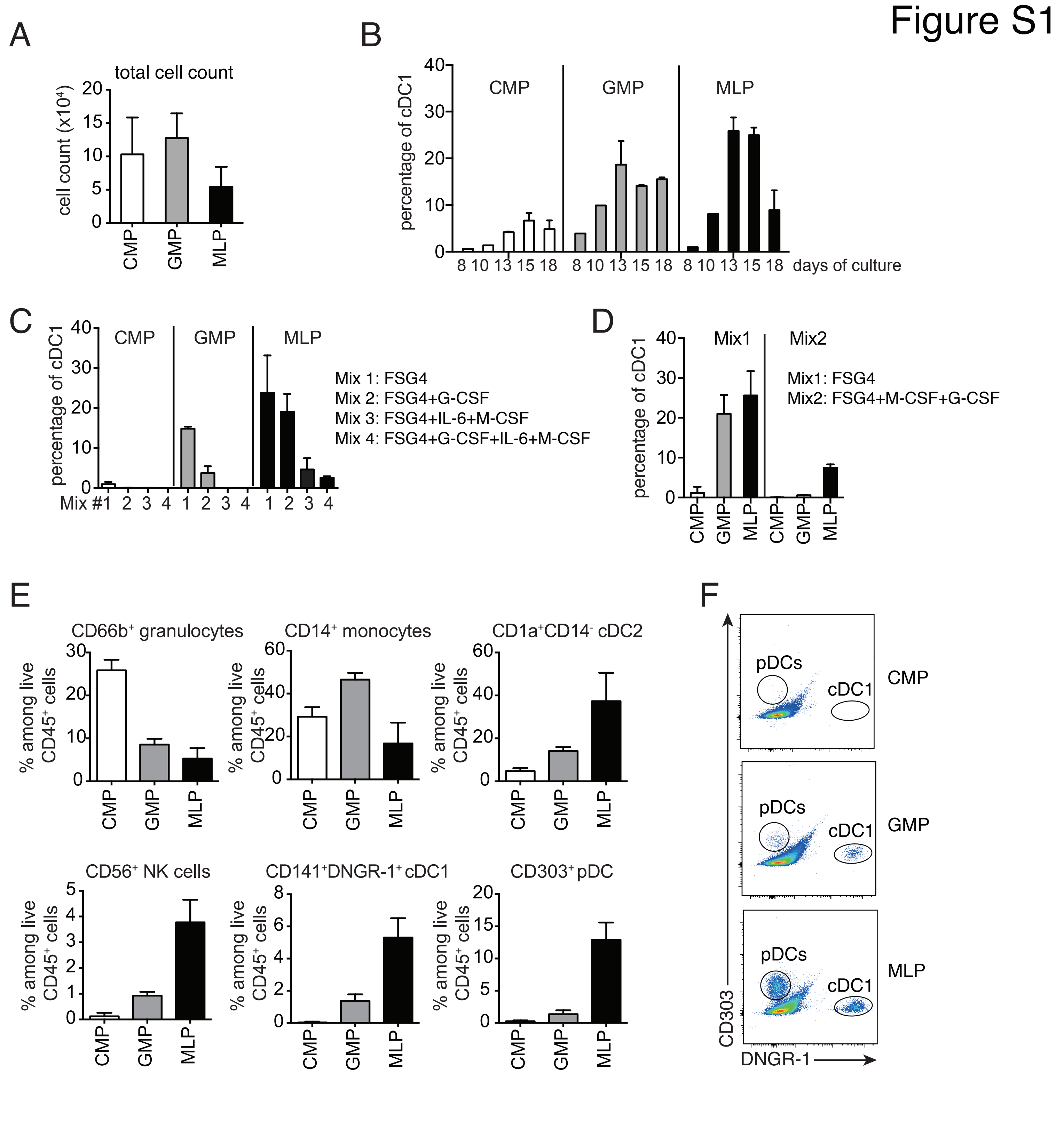

Figure S1. Expansion and cDC1 differentiation potential of CMPs, GMPs and MLPs in vitro. Related to Figure 1.
(A) The indicated progenitors were cultured for 12 days in vitro with FLT3-L, IL-4, SCF and GM-CSF (FSG4). Total cell count was determined by flow cytometry using counting beads. Data are average of 3 replicate wells from one experiment representative of at least 4 experiments. (B) CMPs, GMPs and MLPs were cultured for 8, 10, 13, 15 and 18 days in vitro with FSG4. Percentage of CD141⁺DNGR-1⁺ cDC1s was determined by flow cytometry. Data show one experiment representative of 2 experiments. (C-D) CMPs, GMPs and MLPs were cultured for 12 days with the indicated mixes of cytokines. CD141⁺DNGR-1⁺ cDC1 generation was assessed by flow cytometry. Data are average of 3 replicate wells from one experiment. (E-F) CMPs, GMPs and MLPs were cultured for 15 days with FLT3-L, SCF and GM-CSF (FSG) onto a layer of MS5 fibroblasts. (E) CD141⁺DNGR-1⁺ cDC1, CD1c⁺ cDC2, CD303⁺ pDCs, CD56⁺ NK cells, CD14⁺ monocytes and CD66b⁺ granulocytes generation were assessed by flow cytometry. Data are average of 3 replicate wells from one experiment representative of 2 experiments. (F) DNGR-1⁺ cDC1 and CD303⁺ pDCs differentiation was assessed by flow cytometry. Dot plot show that CD303⁺ pDCs do not express DNGR-1.

Figure S2

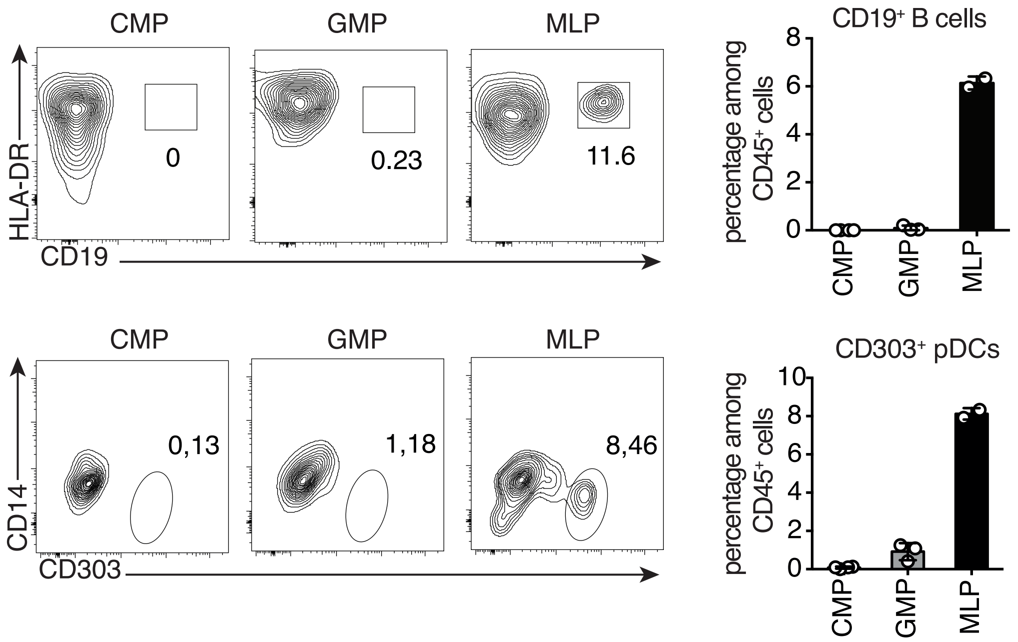

Figure S2. Plasmacytoid DC and B cell production in vivo. Related to Figure 2.
Irradiated NSG-SGM3 mice were injected intravenously with different purified human progenitors. Two weeks later, human B cell and plasmacytoid DC (pDC) presence in the bone marrow was quantitated by flow cytometry. Contour plots show the production of $CD19^{+}$ B cells and $CD303^{+}$ pDCs by the different progenitors. Graphs represent the percentage of each cell type among human $CD45^{+}$ cells engrafted in the bone marrow. Each dot represents an individual mouse, bars indicate the mean and error bars indicate the standard deviation.

Figure S3

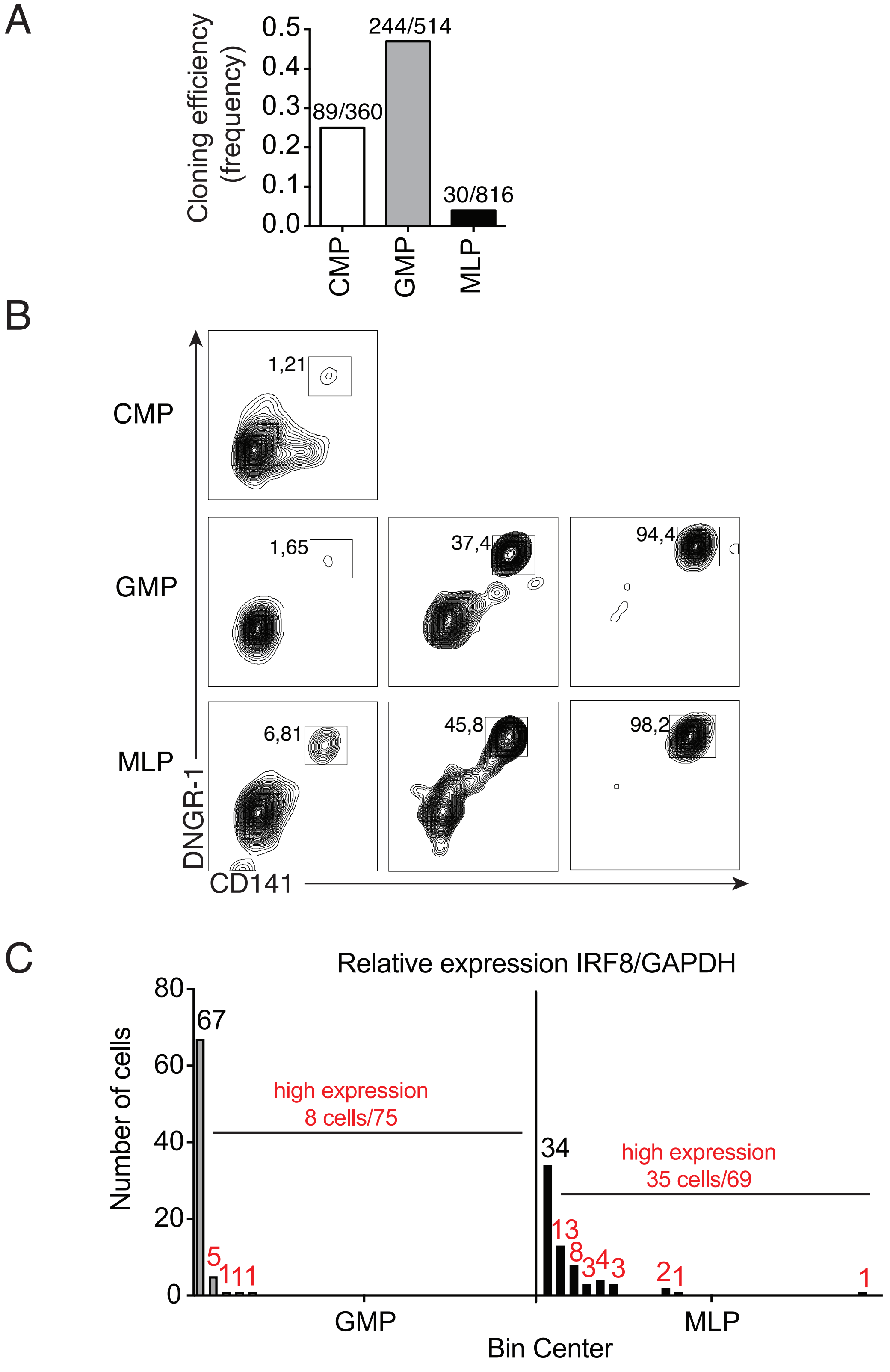

Figure S3. Single cell culture and single cell qPCR. Related to Figure 3.
(A) Cloning efficiency of single cell culture experiments from Figure 3A-C. Graph represents the frequency of successful clones among the total number of seeded wells.
(B) Examples of cDC1 generation in single cell cultures. Dot plots illustrate the % cDC1 that was detected in different wells seeded with CMPs, MLPs or GMPs.
(C) Single progenitors were sorted by flow cytometry into PCR plates (Figure 3D). IRF8 and GAPDH expression was measured for each well by RT-PCR. Bar graphs represent the number of single progenitors expressing different levels of IRF8 relative expression to GAPDH in the same cell. The actual number of cells for each level of expression is indicated on top of each bar. Data are a pool of two independent experiments.

Figure S4

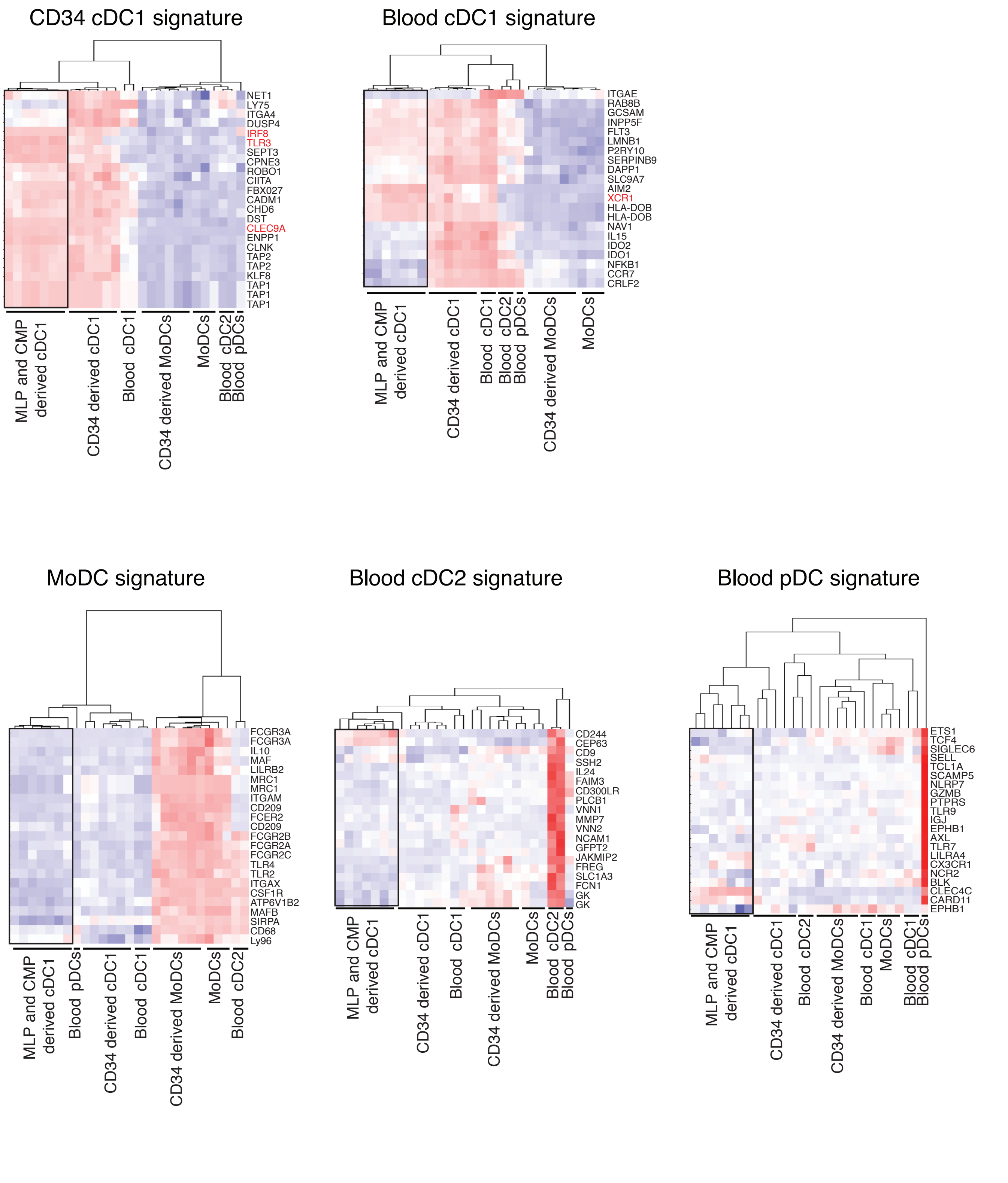

Figure S4. MLP- and CMP-derived cDC1 transcriptomic analysis. Related to Figure 4.
Heat maps of gene expression values comparing our MLP- and CMP-derived cDC1 populations with signatures from a published dataset of cord blood CD34+ cell-derived cDC1 and MoDC, as well as MoDC derived from isolated monocytes and primary cDC1, cDC2 and pDCs from peripheral blood (Balan et al., 2014). Individual replicates are shown.

SUPPLEMENTAL EXPERIMENTAL PROCEDURES

Flow cytometry analysis

Anti-HLA-DR (G46.6), anti-CD34 (8G12), anti-CD10 (HI10a), anti-CD19 (HIB19), and anti–Lineage-1 cocktail (CD3, CD14, CD16, CD19, CD20, and CD56) were purchased from BD. Anti-CD123 (6H6), anti-CD45RA (HI100), anti-CD38 (HIT2), anti-CD45 (2D1), anti-CD11c (3.9), anti-CD14 (61D3) were purchased from eBioscience. Anti-DNGR-1 (8F9) and purified isotype control were purchased from Biolegend. Anti-BDCA-1/CD1c (AD5-8E7) and anti-BDCA-3/CD141 (AD5-14H12) were purchased from Miltenyi Biotec. Isotype-matched irrelevant specificity control mAbs were used to control for unspecific staining.

Single cell qPCR

For cDNA production, cells were lysed in 7.2µl of a mix containing 0.51µl of NP40 10%, 0.37µl DTT, 0.15µl of rRNasin (Promega) and 1.03µl of random hexamer primers (Applied Biosystems), during 1 minute at 65°C. Then cDNA was synthesized from total RNA by adding 7µl of a second mix containing: 0.5µl of dNTP (Roche), 1µl DTT, 0.2µl rRNasin (Promega) and 0.3µl Superscript III (Invitrogen). Plate was incubated for 15 minutes at room temperature and then RT-PCR reaction was performed as follows: 5 minutes at 42°C, 10 minutes at 25°C, 55 minutes at 50°C and 5 minutes at 94°C.

Microarray analysis

Data were analysed using Bioconductor 2.13 (http://www.bioconductor.org) running on R 3.0.2 (available from www.R-project.org). Probeset expression measures were calculated using the Affymetrix package's Robust Multichip Average (RMA) default method. Differentially expressed genes were assessed between samples using an empirical Bayes t-test (limma package). P values were adjusted for multiple testing using the Benjamini-Hochberg method. Any probe sets that exhibited an adjusted P value of 0.05 were called differentially expressed. Two-dimensional hierarchical clustering of expression data was carried out using a 1 − Pearson correlation distance matrix and average linkage clustering. Genes were clustered using a Euclidean distance matrix. For heat map analysis, colors indicate the expression value relative to the mean expression value per gene in the dataset. Red indicates upregulation and blue indicates downregulation relative to mean value.
