# Supplementary material for: Dendritic Cell Lineage Potential in Human Early Hematopoietic Progenitors
Source: Cell Rep. 2017 Jul 18;20(3):529–37. doi: 10.1016/j.celrep.2017.06.075 (PMC5529209; doi:10.1016/j.celrep.2017.06.075)
Supplement: Document S2. Article plus Supplemental Information [file mmc2.pdf]

Cell Reports

Report

Dendritic Cell Lineage Potential in Human Early Hematopoietic Progenitors

Graphical Abstract

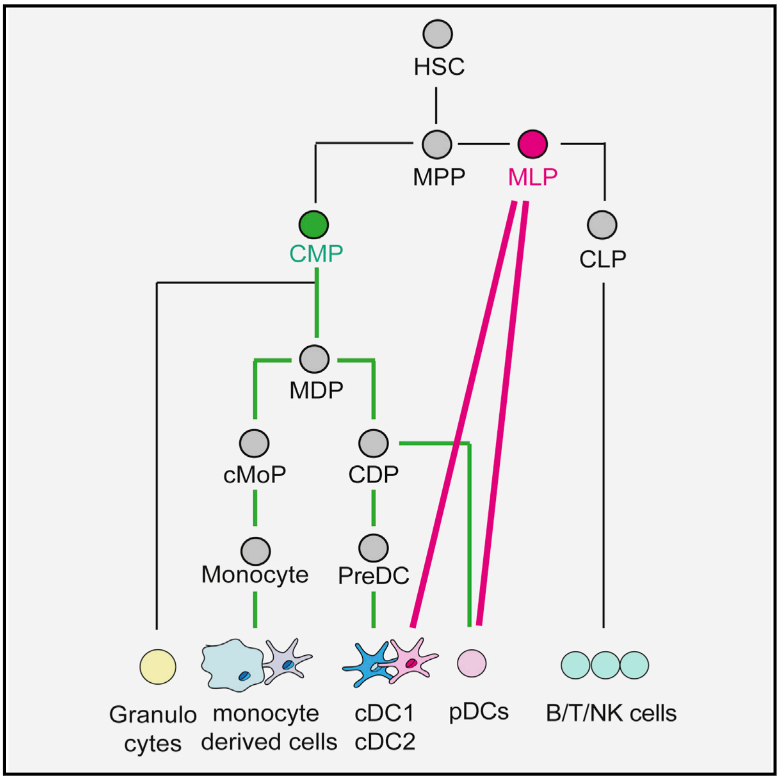

Highlights

- MLPs generate human CD141+DNGR-1+ cDC1s more efficiently than CMPs
- Single-cell culture and qPCR reveal MLPs biased toward cDC1 differentiation
- cDC1s derived from MLPs are identical to those derived from CMPs

Authors

Julie Helft, Fernando Anjos-Afonso, Annemarthe G. van der Veen, Probir Chakravarty, Dominique Bonnet, Caetano Reis e Sousa

Correspondence

julie.helft@curie.fr (J.H.), caetano@crick.ac.uk (C.R.e.S.)

In Brief

Dendritic cells (DCs) are thought to descend from a DC precursor downstream of the common myeloid progenitor (CMP). Helft et al. show that multipotent lymphoid progenitors (MLPs) in humans are more efficient producers of CD141^{+}DNGR-1^{+} cDC1s than CMPs. Therefore, DC lineage imprinting can occur in early hematopoietic progenitors in humans.

Accession Numbers

GSE98957

Helft et al., 2017, Cell Reports *20*, 529–537
July 18, 2017 © 2017 The Author(s).
http://dx.doi.org/10.1016/j.celrep.2017.06.075

Cell Reports
Report

OPEN ACCESS
CellPress

Dendritic Cell Lineage Potential in Human Early Hematopoietic Progenitors

Julie Helft,[1,5,6,*] Fernando Anjos-Afonso,[2,4] Annemarthe G. van der Veen,[1] Probir Chakravarty,[3] Dominique Bonnet,[2] and Caetano Reis e Sousa[1,*]
[1]Immunobiology Laboratory
[2]Haematopoietic Stem Cell Laboratory
[3]Bioinformatics
The Francis Crick Institute, 1 Midland Road, London NW1 1AT, UK
[4]Cardiff University, European Cancer Stem Cell Institute, Cardiff, CF10 3XQ, UK
[5]Present address: Institut Curie, PSL Research University, INSERM U932, 75005 Paris, France
[6]Lead Contact
*Correspondence: julie.helft@curie.fr (J.H.), caetano@crick.ac.uk (C.R.e.S.)
http://dx.doi.org/10.1016/j.celrep.2017.06.075

SUMMARY

Conventional dendritic cells (cDCs) are thought to descend from a DC precursor downstream of the common myeloid progenitor (CMP). However, a mouse lymphoid-primed multipotent progenitor has been shown to generate cDCs following a DC-specific developmental pathway independent of monocyte and granulocyte poiesis. Similarly, here we show that, in humans, a large fraction of multipotent lymphoid early progenitors (MLPs) gives rise to cDCs, in particular the subset known as cDC1, identified by co-expression of DNGR-1 (CLEC9A) and CD141 (BDCA-3). Single-cell analysis indicates that over one-third of MLPs have the potential to efficiently generate cDCs. cDC1s generated from CMPs or MLPs do not exhibit differences in transcriptome or phenotype. These results demonstrate an early imprinting of the cDC lineage in human hematopoiesis and highlight the plasticity of developmental pathways giving rise to human DCs.

INTRODUCTION

Dendritic cells (DCs) are mononuclear phagocytes crucial for the initiation and regulation of immune responses (Steinman et al., 2003). They are classically divided into plasmacytoid DCs (pDCs) and two distinct subsets of conventional DCs (cDCs), termed cDC1 and cDC2 (Guilliams et al., 2014). The cDC1 subset constitutes a homogeneous cell population identified by surface expression of the chemokine receptor XCR1 and the C-type lectin DNGR-1 (also known as CLEC9A) in both mice and humans (Crozat et al., 2010; Poulin et al., 2012) and is defined by its developmental dependence on the transcription factors BATF3 and IRF8 (Aliberti et al., 2003; Hildner et al., 2008; Murphy et al., 2016) and the growth factors fms-like tyrosine kinase 3 ligand (FLT3L) and granulocyte-macrophage colony-stimulating factor (GM-CSF) (Ginhoux et al., 2009; Greter et al., 2012; McKenna et al., 2000; Merad et al., 2013). cDC1s play a prominent role in cross-presentation of dead cell-associated antigens and in Th1 and cytotoxic T lymphocyte priming (Merad et al., 2013). cDC2s are $CD11b^+$ $CD172a^+$, and their differentiation depends on IRF4, IRF2, TRAF6, RelB, and RBP-J transcription factors (Murphy et al., 2016). However, mouse $CD11b^+$ cDC2s are considerably heterogeneous and include a subtype whose differentiation depends on KLF4 and induces Th2-dominated immunity (Tussiwand et al., 2015) as well as gut $CD103^+CD11b^+$ DCs that prominently induce Th17 responses against pathobionts (Persson et al., 2013; Schlitzer et al., 2013). Human cDC2s are often identified as $HLA\text{-}DR^+$ $CD11c^+$ $CD11b^+$ $CD1a^+$ $CD1c^+$ leukocytes (Caux et al., 1996; Doulatov et al., 2010) with variable expression of CD14 (Lee et al., 2015). As for mice, human cDC2 are heterogeneous (Villani et al., 2017) and, in some cases, can be contaminated with monocyte progeny that is referred to as "monocyte-derived DCs" and has a similar surface phenotype (McGovern et al., 2014). Additional DC subtypes have very recently been described in human blood, suggesting that DC heterogeneity may be even greater than previously appreciated (Villani et al., 2017).

DCs are derived from hematopoietic progenitors that are continuously produced in adult bone marrow by hematopoietic stem cells (HSCs) (Merad et al., 2013). Early studies indicated that mouse and human common myeloid progenitors (CMPs) and common lymphoid progenitors (CLPs) both have the potential to generate DCs, suggesting that DCs can be generated by either myelopoiesis or lymphopoiesis (Chicha et al., 2004; Karsunky et al., 2003; Manz et al., 2001; Traver et al., 2000). However, a fate-mapping experiment using IL7Ra-Cre excluded a significant contribution of lymphoid progenitors to DC generation and placed DCs squarely within the myelopoietic branch (Schlenner et al., 2010). Consistent with that notion, a current view of DC development is that CMPs give rise to macrophage/DC progenitors (MDPs), first identified in mice and then in humans (Fogg et al., 2006; Lee et al., 2015), which further differentiate into a common DC progenitor (CDP) that is no longer able to generate monocytes (Lee et al., 2015; Naik et al., 2007; Onai et al., 2007). In turn, CDPs give rise to circulating pre-DCs that leave the bone marrow and travel via the blood to seed

This is an open access article under the CC BY license (http://creativecommons.org/licenses/by/4.0/).

lymphoid and non-lymphoid organs, giving rise to differentiated DCs (Breton et al., 2015; Ginhoux et al., 2009; Liu et al., 2009; See et al., 2017). This model of DC development supports a classical view of hematopoiesis where DC specification occurs through stepwise loss of multi-lineage potential by myeloid progenitors (Akashi et al., 2000; Kondo et al., 1997; Reya et al., 2001).

That model was challenged by a barcoding study that analyzed the progeny of mouse early lymphoid multipotent primed progenitors (LMPPs) (Naik et al., 2013). It found that a large proportion of LMPPs is already imprinted with the potential to give rise to cDCs independently of monocytes or granulocytes. In addition, a re-analysis of putative mouse MDPs found that only a very small fraction of cells was truly bi-potential at the clonal level (Sathe et al., 2014). Altogether, these findings would seem to indicate that the differentiated mouse cDC pool might reflect a mixed contribution of MDPs as well as cDC-imprinted LMPPs, as argued for the pDC lineage (Shortman et al., 2013). Similarly, in humans, early lympho-myeloid progenitors might contribute to DC generation because the multipotent lymphoid progenitor (MLP; Lin^{-}CD34^{+}CD38^{-}CD45RA^{+}CD10^{+}) can generate monocytes and HLA-DR^{+} CD1a^{+} CD11c^{+} CD11b^{+} cells in addition to all lymphoid cells (Doulatov et al., 2010). However, it remains unclear whether the HLA-DR^{+} CD1a^{+} CD11c^{+} CD11b^{+} cells generated by human lympho-myeloid progenitors correspond to bona fide conventional cDC2 or, instead, reflect a monocyte-derived DC. A more stringent test of the cDC-generating potential of MLPs would be to assess whether the precursor population can give rise to cDC1, but this has not been reported. Finally, it is unclear whether different DCpoietic pathways, if they exist, would give rise to identical cells. Here we tested the potential of CMPs versus MLPs to generate human cDC1 and cDC2 cells. We report that human MLPs can efficiently generate both cDC1s and cDC2s and that MLP- and CMP-derived cDC1 cells are transcriptionally indistinguishable. These results support a model in which specification of the cDC lineage can occurs early in hematopoiesis in humans and underscore the diversity of hematopoietic decisions giving rise to identical human DCs.

RESULTS

MLPs, CMPs, and GMPs Can Generate cDCs In Vitro and In Vivo

We have previously shown that unfractionated CD34^{+} umbilical cord blood hematopoietic cells can be differentiated in vitro into CD1a^{+}HLA-DR^{+}CD141^{+}DNGR-1^{+} cDC1s (Poulin et al., 2010, 2012) under the aegis of FLT3-L, stem cell factor (SCF), GM-CSF, and interleukin-4 (IL-4) (FSG4). This culture condition also allows the differentiation of CD1c^{+} cDC2 cells and of CD14^{+} monocytes (Balan et al., 2014; see below). To analyze the actual origin of DCs developing in FSG4 cultures, we isolated different hematopoietic progenitors from human cord blood by flow cytometry (Figure 1A) following established protocols (Akashi et al., 2000; Chicha et al., 2004; Doulatov et al., 2010) to sort MLPs, CMPs, and granulocyte/macrophage progenitors (GMPs), which originate from CMPs but are now known to also overlap in phenotype with DC precursors (Lee et al., 2015). MLPs, CMPs, and GMPs all express FLT3 (Doulatov et al., 2010; Lee et al., 2015), allowing them to respond to FLT3L, a key pre-requisite for DC differentiation (D'Amico and Wu, 2003). Sorted MLP, CMP, and GMP populations were then cultured with FSG4, and their DC- and monocyte/macrophage (mono/mac)-generating potential was analyzed.

We found that MLPs, CMPs, and GMPs were all able to expand in the FSG4 culture system (Figure S1A) and give rise to CD141^{+}DNGR-1^{+} cDC1, CD1a^{+}CD1c^{+}CD14^{-} cDC2, and CD1a^{-}CD14^{+} mono/macs (Figure 1B). MLPs were efficient cDC1 progenitors both in terms of percentage and number of CD141^{+}DNGR-1^{+} cells generated and were less efficient at generating cDC2s by the same criteria (Figure 1B). GMPs were similar in efficiency to MLPs at generating CD141^{+}DNGR-1^{+} cDC1s, but, intriguingly, CMPs only gave rise to modest numbers of the same DCs. However, CMPs were as efficient as MLPs at generating cDC2s and mono/macs in the FSG4 culture system (Figure 1B). We tested whether the different progenitors produced CD141^{+}DNGR-1^{+} cDC1s with different kinetics. We found that CMPs, GMPs, and MLPs followed the same kinetics of differentiation into CD141^{+}DNGR-1^{+} cDC1s, with a peak around day 13 in FSG4 (Figure S1B). We then tested whether the low cDC1-generating potential of CMPs could be rescued by adding other cytokines to the FSG4 cocktail. All cytokines tested (IL-6, M-CSF, and G-CSF) extinguished rather than improved cDC1 generation by CMPs (Figures S1C and S1D). Finally, we tested the lineage potential of the progenitors in a more permissive cytokine environment comprising FLT3-L, SCF, and GM-CSF (FSG) (Figure S1E). This cytokine cocktail enables the differentiation of all DC subsets (cDC1s, cDC2s, and pDCs) as well as natural killer (NK) cells, monocytes, and granulocytes (Lee et al., 2015). Similar to FSG4 cultures, MLP was the most efficient producer of CD141^{+}DNGR-1^{+} cDC1s. MLP was also the only progenitor giving rise to CD56^{+} NK cells and to CD303^{+} pDCs, which were phenotypically distinct from DNGR-1^{+} cDC1s (Figures S1E and S1F). In contrast, CMPs and GMPs were more efficient at generating CD14^{+} monocytes and CD66b^{+} granulocytes (Figure S1E). Altogether, these results show that MLPs are the most efficient cDC1 progenitors in various culture settings.

To confirm these results in vivo, we transferred MLPs, CMPs, and GMPs into non-obese diabetic (NOD)/severe combined immunodeficiency (SCID)/IL-2Rγ-null/Tg(CMV-IL3, CSF2, KIT ligand [KITLG]) (NSG-SGM3) mice (Billerbeck et al., 2011). Two weeks later, DC content was analyzed in the bone marrow of recipients. In mice receiving MLPs, CD141^{+}DNGR-1^{+} cDC1s represented around 40% of all human CD45^{+} cells, whereas, in CMP recipients, this only reached 10% (Figure 2). In contrast, all progenitors gave rise to a similar low frequency of cDC2s, whereas GMPs were slightly more efficient than CMPs or MLPs at generating CD14^{+} mono/macs. As expected, only MLPs could generate CD19^{+} B cells, confirming their lymphoid potential (Figure S2). Interestingly, MLPs were also the only progenitors able to give rise to pDCs (Figure S2). Together, these results show that, at bulk population level, CMPs, GMPs, and MLPs can all give rise to cDC1s, cDC2s, and mono/macs. CMPs appear to be biased toward cDC2 and mono/mac generation, whereas MLPs produce relatively more cDC1s and pDCs both in vitro

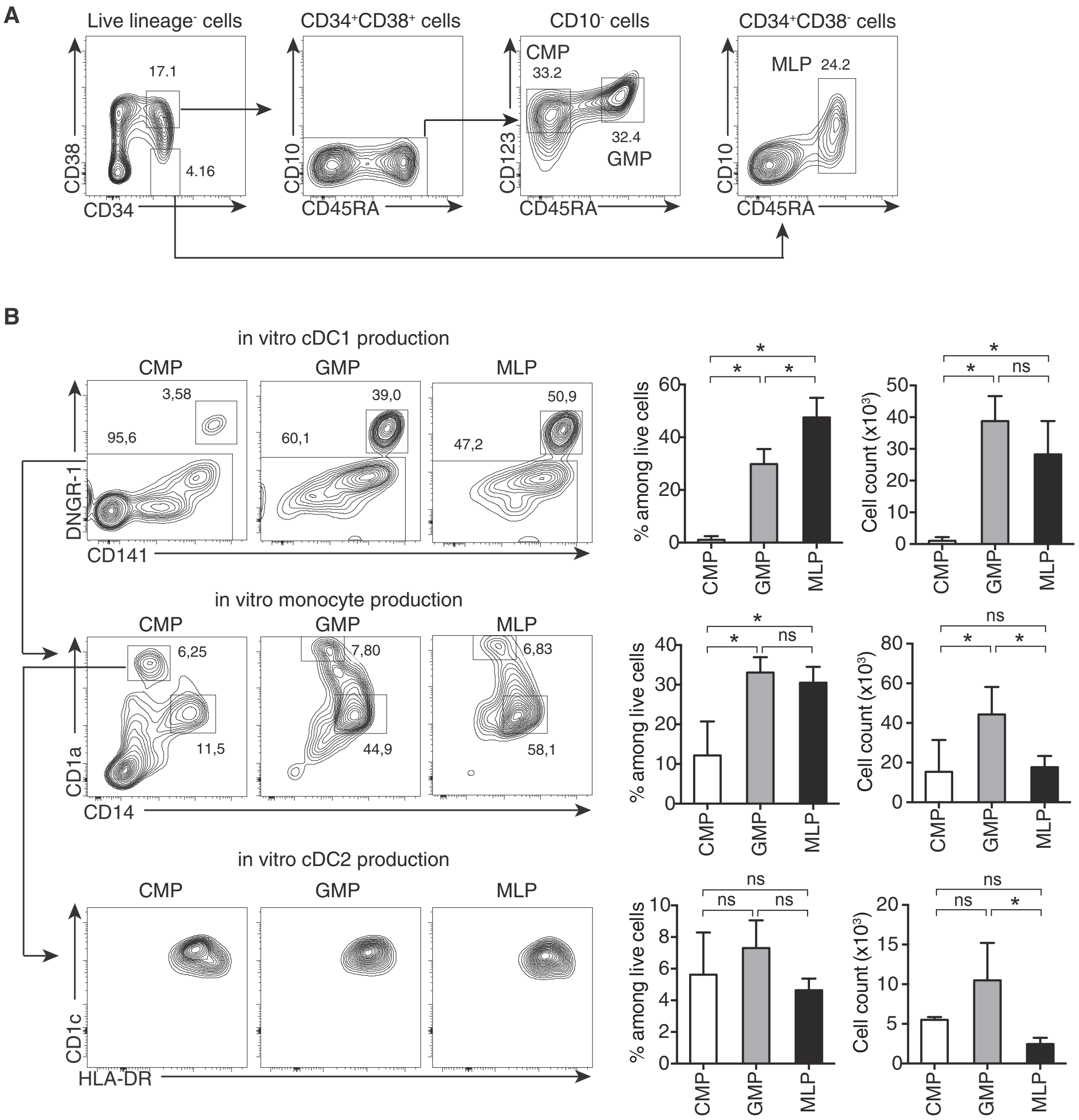

Figure 1. MLPs, CMPs, and GMPs Can Generate cDCs In Vitro

(A) Gating strategy for human hematopoietic progenitors isolated from umbilical cord blood. Boxes depict gates, and numbers correspond to the percentage of cells in each gate.

(B) 500 of the indicated progenitor cells isolated according to the gating strategy in (A) were cultured for 12 days in vitro with FLT3-L, IL-4, SCF, and GM-CSF. $CD141^+DNGR\text{-}1^+$ cDC1, $CD14^+CD1a^-$ mono/mac, and $CD14^-CD1a^+CD1c^+$ cDC2 progeny were analyzed by flow cytometry. Contour plots depict the gating strategy used to identify cDC1s, cDC2s, and mono/macs. Graphs show the percentage (left) and numbers (right) of each phagocyte subtype produced and are the average of three independent cultures. Error bars depict SD. * $p < 0.05$ (one-way ANOVA). Data are from one experiment representative of at least four independent experiments.

and in vivo. Thus, commitment to the human cDC lineage can occur in early hematopoietic progenitors with myeloid or lympho-myeloid potential in various experimental settings. Importantly, MLPs present the best potential for cDC1 and pDC production compared with CMPs and GMPs.

Single-Cell Potential of DC Progenitors

To assess the potential of the different progenitors to generate mononuclear phagocytes at the single-cell level, we set up an in vitro clonal assay in which single MLPs, GMPs, or CMPs were isolated and cultured with MS5 stromal cells and FSG4 for 12 days (see Figure S3A for cloning efficiency). The ability to generate cDC2s and mono/macs did not differ strikingly among different progenitors and was found in 40%–60% of the starting populations (Figure 3A). This confirms that DC- and mono/mac-generating potential is not restricted to a small fraction of contaminating cells in any of the populations. The cloning efficiency of MLPs was low, which could be due to an effect of

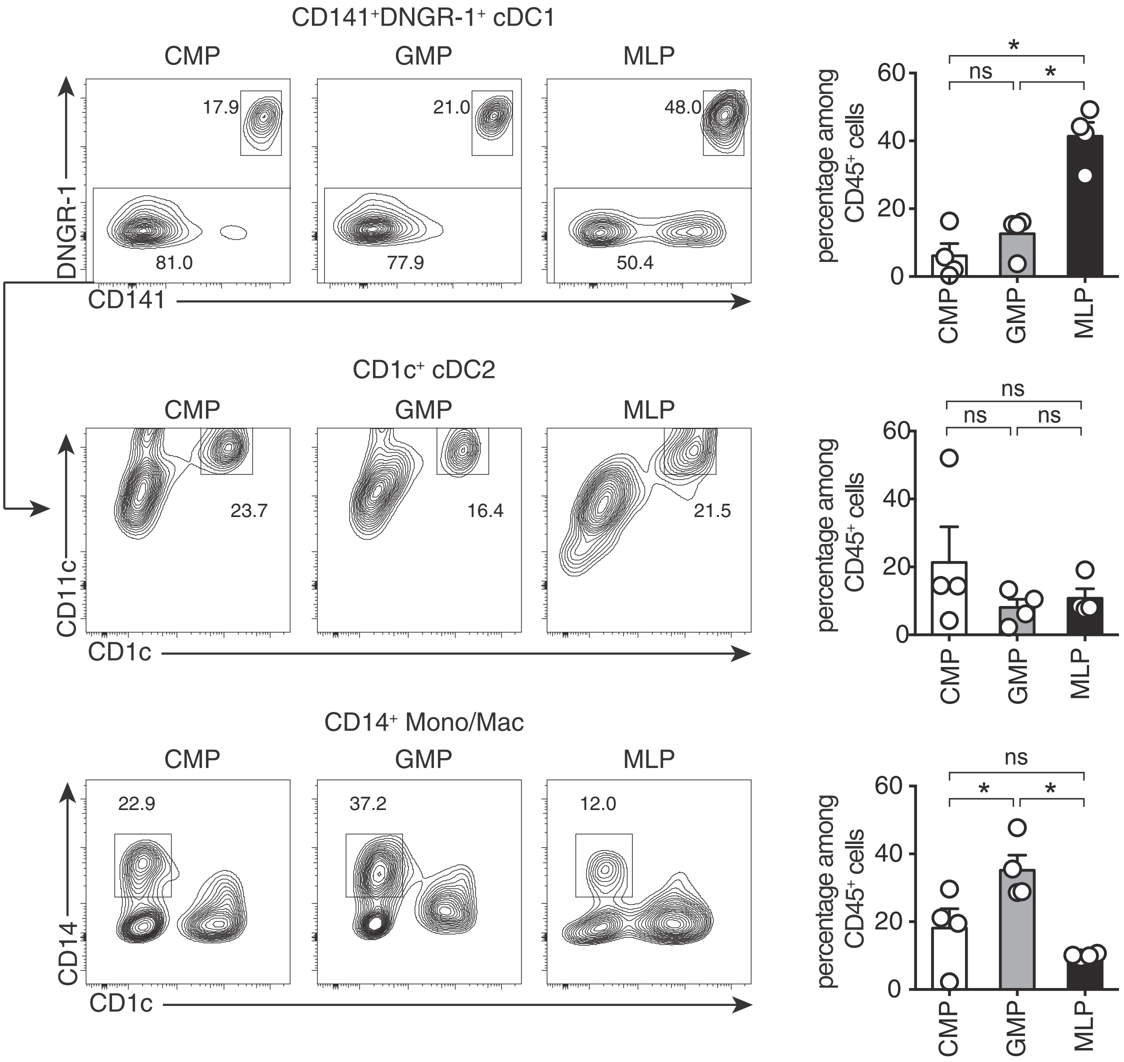

Figure 2. MLPs, CMPs, and GMPs Can Generate cDCs In Vivo

Irradiated NSG-SGM3 mice were injected intravenously with different human progenitors. Two weeks later, human cDC1, cDC2, and monocyte presence in the bone marrow was quantitated by flow cytometry. Contour plots and graphs show the generation of CD141$^+$DNGR-1$^+$ cDC1, CD14$^-$CD11c$^+$CD1c$^+$ cDC2, and CD14$^+$CD1c$^-$ cells by the different progenitors. Each dot represents an individual mouse, bars indicate the mean, and error bars indicate SD. *$p < 0.05$ (one-way ANOVA). Data are a pool of two independent experiments.

combining FSG4 with MS5 without re-optimizing the cytokine cocktail (unpublished observations). Nevertheless, we found that more than 50% of the MLPs that generated detectable clones in MS5 + FSG4 cultures could give rise to cDC1s (Figure 3A). Moreover, a higher frequency of MLPs could generate cDC2s than give rise to mono/macs (Figure 3A), and approximately 37% of single MLPs could differentiate into cDCs (cDC1s and cDC2s) without generating other myeloid cells (Figure 3B). This was not the case for the CMPs, which never generated cDC-only progeny (Figure 3B). These findings suggest that some hematopoietic progenitors are pre-imprinted with the potential to give rise to cDCs independently of mono/mac. To explore this notion, we first focused on the cDC1 subset, which is more homogeneous than cDC2 (Villani et al., 2017) and, therefore, a reliable indicator of cDC commitment. The percentage of cDC1s generated by individual MLPs or GMPs varied between 1% and 99% (Figure 3C, top; Figure S3B), which suggests the presence inside of MLP and GMP populations of a spectrum of cells that have global myeloid potential versus cDC-only potential. In contrast, the percentage of cDC1s generated by individual CMPs never reached more than 10%, confirming the absence of clones with a cDC-only potential (Figure 3C, top; Figure S3B). We then analyzed cDC2 generation efficiency under the same conditions. Similar to cDC1 generation, the percentage of cDC2s generated by individual MLPs and GMPs varied between 1% and 99% (Figure 3C, bottom). Interestingly, CMPs were more efficient on a per-cell basis at generating cDC2s than cDC1s because some CMP clones could generate more than 50% of cDC2s. This could suggest that CMPs and MLPs generate different subsets of cDC2s (Villani et al., 2017), although this

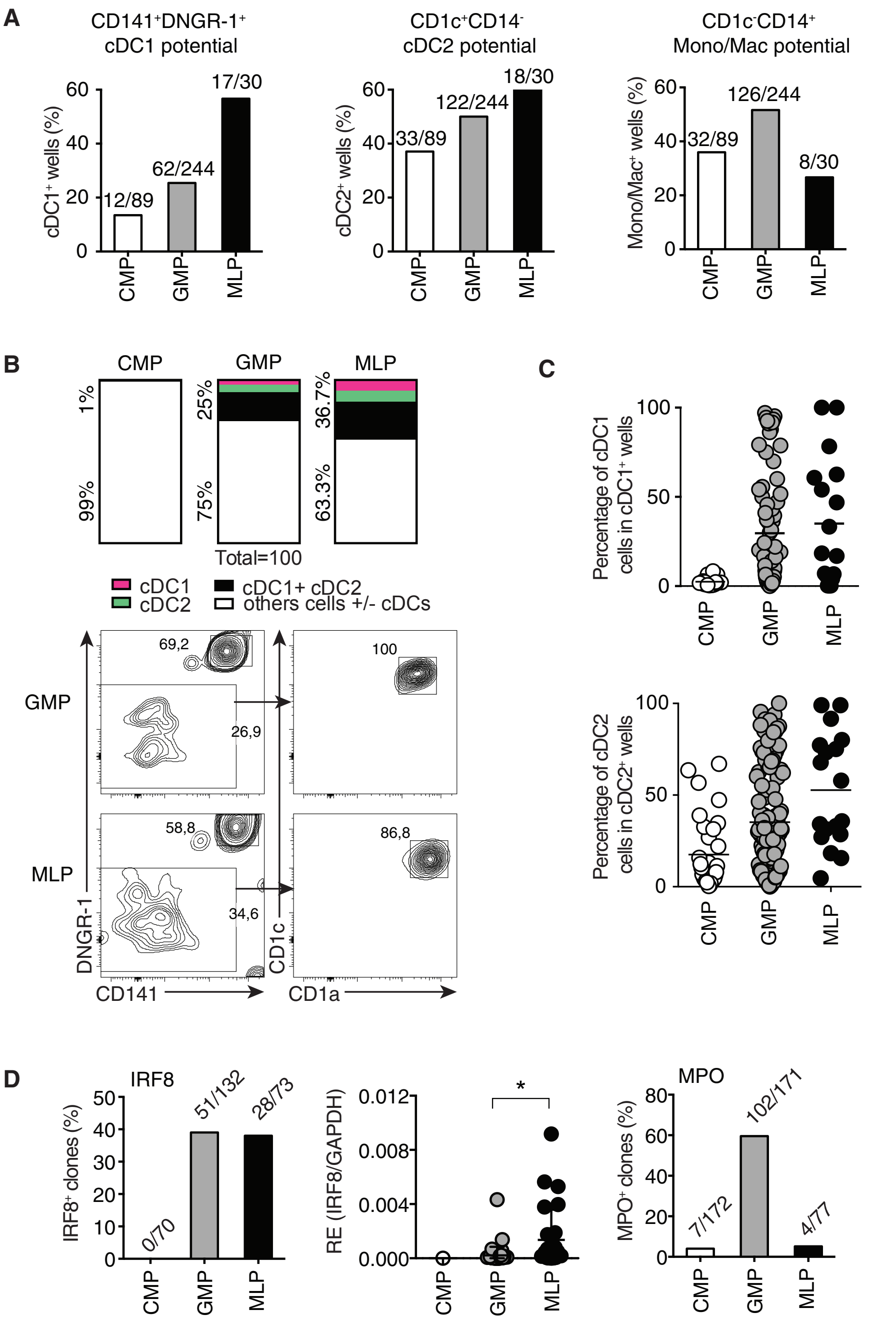

Figure 3. Single-Cell Potential of DC Progenitors

(A) Single progenitor cells were deposited on a layer of MS5 cells and cultured for 12 days with FLT3-L, IL-4, SCF, and GM-CSF. cDC1, cDC2, and mono/mac presence in each well was analyzed by flow cytometry. Bars represent the percentage of wells that contained each of the indicated populations irrespective of the presence or absence of any others. The actual number of wells is indicated on top of each bar. Data are a pool of four independent experiments.

(B) Bar graph showing the percentage of single progenitors producing only cDC1 cells (pink), only cDC2 cells (green), or only cDC1 and cDC2 cells (black). White includes wells that gave rise to other cell types with or without cDCs. Contour plots show an example for single GMP or MLP culture wells containing only cDC1 and cDC2.

(C) cDC1 and cDC2 generation in single-cell cultures. The graphs illustrate the percentage of cDC1 (top) or % of cDC2 (bottom) detected in each cDC1- or cDC2-positive well seeded with single CMPs, MLPs, or GMPs. The data are a pool of four independent experiments. The lines represent the mean.

(D) Bar graphs representing the percentage of IRF-8$^+$ (left) or MPO$^+$ (right) single progenitor cells among total GAPDH$^+$ cells, as determined by single-cell qRT-PCR. The actual number of IRF-8$^+$ or MPO$^+$ cells compared with the total number of GAPDH$^+$ cells is indicated on top of each bar. The center graph shows the relative expression (RE) of IRF8 compared with GAPDH for each IRF8-positive cell. *$p < 0.001$ represents statistically significant differences in expression between GMPs and MLPs (unpaired t test). Data are from one experiment representative of two independent experiments.

was not assessed. Consistent with their cDC-restricted potential, about 40% of MLPs and GMPs, but no CMPs, expressed, at the single-cell level, mRNA for *IRF8* (Figure 3D), the key cDC1-specifying factor. In addition, among MLPs and GMPs expressing *IRF8*, 50% of MLPs but only 10% of GMPs had higher levels of *IRF8* mRNA per cell (Figure 3D; Figure S3C). Finally, only 5% of MLP clones expressed mRNA for myeloperoxidase (MPO), a marker of myeloid commitment that was found in over 50% of GMPs (Figure 3D). Altogether, these data indicate that early and multipotent lymphoid-primed progenitors such as MLPs, but not myeloid progenitors such as CMPs, contain cells with high potential for cDC generation that can even give rise to a single cDC subset (cDC1).

MLP- and CMP-Derived cDC1s Are Transcriptionally Identical

Although cDC1s are thought to be homogeneous, the finding that CD1a$^+$HLA-DR$^+$CD141$^+$DNGR-1$^+$ cells could be generated from MLPs (efficiently) or CMPs (less efficiently) prompted the question of whether they are the same cells. We therefore carried out a transcriptomic analysis of MLP- or CMP-derived cDC1s and compared both profiles with a published dataset of DC subsets and monocyte-derived DC (MoDCs) generated in vitro from total CD34$^+$ HSCs or purified from peripheral blood (Balan et al., 2014). We found that both MLP- and CMP-derived cDC1s expressed the classical cDC1 gene signature, which includes, among others, *IRF8*, *TLR3*, *CLEC9A*, and *XCR1* transcripts (Figure 4A; Figure S4). We could also confirm that MLP- and CMP-derived cDC1 did not express any of the signature genes of MoDCs or pDCs

OPEN ACCESS
CellPress

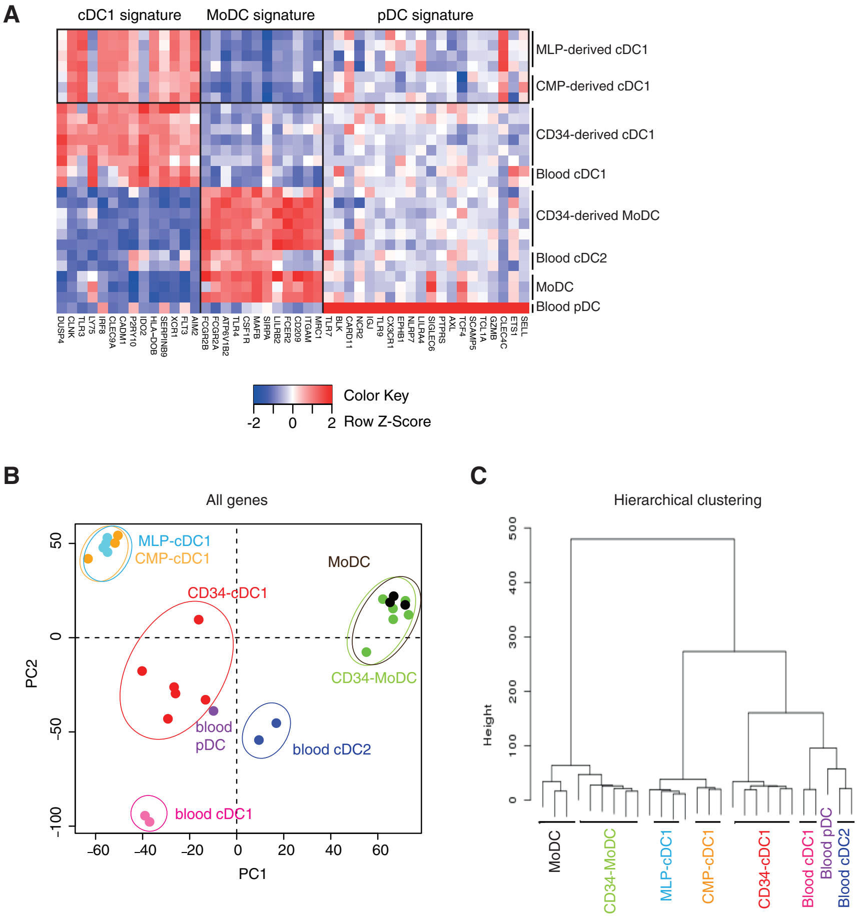

Figure 4. MLP- and CMP-Derived cDC1 Transcriptomic Analysis

(A) Heatmap of gene expression values comparing MLP- and CMP-derived cDC1 populations with a published dataset (GSE57671) of cord blood CD34^{+} cell-derived cDC1s and MoDCs as well as MoDCs derived from purified blood monocytes and primary cDC1, cDC2, and pDCs purified from peripheral blood (Balan et al., 2014). Individual replicates are shown.

(B) Principal component analysis of all genes expressed in MLP- and CMP-derived cDC1 cells and in DC populations described in Balan et al., 2014. Each dot of the same color corresponds to a replicate sample.

(C) Hierarchical clustering of triplicate samples of MLP- and CMP-derived cDC1s and published dataset of cord blood CD34^{+} cell-derived cDC1s and MoDCs as well as MoDCs derived from blood monocytes and primary blood cDC1s, cDC2s, and pDCs (Balan et al., 2014). The 2% of genes with the most variable expression were used for the analysis.

(Figure 4A; Figure S4). We then compared MLP- or CMP-derived cDC1s with each other by principal component analysis. This revealed that MLP- and CMP-derived cDC1s clustered tightly together (Figure 4B) and did not display any statistically significant differences in gene expression (data not shown). As expected, MLP- and CMP-derived cDC1s were closest to cDC1 produced in vitro from CD34^{+} HSC/progenitors or purified from human blood (Figure 4B). This was confirmed by unsupervised hierarchical clustering using the 2% of genes with the most variable expression (Figure 4C). We conclude that MLP- and CMP-derived CD141^{+}DNGR-1^{+} cells are indistinguishable and represent phenotypically bona fide cDC1s.

DISCUSSION

DCpoiesis is often thought to constitute a branch of myelopoiesis. Our study shows that human cDC progenitors are enriched within the pool of early hematopoietic progenitors, the MLPs, that gives rise to lymphoid cells. This result mirrors a recent study in mice that used barcoding to follow in vivo the cellular output of single LMPPs and found that 50% of the cells were imprinted toward the cDC lineage (Naik et al., 2013). In contrast, another study has recently identified a human MDP in the CD34^{+} fraction of human umbilical cord blood and bone marrow, consistent with the classical view that DCs derive from a myeloid branch-producing progenitors with increased commitment toward the DC lineage (Lee et al., 2015). However, Lee et al. (2015) show that only 13% of single MDPs are able to generate both cDCs and monocytes (Lee et al., 2015). Similarly, in mice, the bi-potentiality of single MDPs is found in a small fraction of cells (Sathe et al., 2014). Therefore, bi-potent monocyte/cDC progenitors may co-exist with CDPs that derive directly from MLPs and do not have an MDP ancestor.

In humans, the phenotype of the GMP overlaps partially with that of DC precursors (Lee et al., 2015; See et al., 2017). The GMP population is therefore likely to be heterogeneous and contain a substantial fraction of DC precursors, unlike the CMP population (Lee et al., 2015), explaining why GMPs appear to be more efficient than their CMP progenitors at generating DCs. In contrast, DC progenitors do not overlap in phenotype with MLPs, and our findings of efficient DC generation by MLPs cannot be ascribed to a small sub-fraction of contaminating cells because cDC-generating potential was present in more than 50% of cloneable MLPs. This is consistent with the fact that around half of all MLPs express IRF8, a transcription factor that has been shown to auto-activate and trigger cDC1 subset differentiation (Grajales-Reyes et al., 2015) and the loss of which leads to human DC deficiency (Hambleton et al., 2011). Therefore, as in mice, human DCs appear to have two types of progenitors. One "late" progenitor shared with monocytes (Fogg et al., 2006; Lee et al., 2015) and one found very early in the hematopoietic tree at the MLP level (Naik et al., 2013). Which of these progenitors contributes most to the steady-state pool of cDCs is unknown.

These results, suggesting a dual ontogeny of cDCs, led us to ask whether cDC1 cells deriving from distinct developmental

pathways are equivalent. The latter would indicate an overriding role of "nurture" in cDC differentiation, whereas the former would suggest that cellular "nature" leaves an indelible imprint in progeny at the level of gene expression and, perhaps, function. Interestingly, we found an exclusive role for nurture in that MLP-derived and CMP-derived cDC1s possess identical transcriptomes. Because gene expression underlies cell function, we presume that both sources of cDC1s lead to cells with identical properties, although we did not perform exhaustive functional analyses. Interestingly, mouse pDCs can also originate from either myelo- or lymphopoietic branches and are seemingly identical, other than displaying or not displaying a history of recombination activating gene (RAG) expression (Sathe et al., 2013; Shortman et al., 2013). Whether distinct pathways of DC production prevail in different settings remains to be explored, as does the possibility that, under some circumstances, cDCs derived from MLPs and those derived from MDPs may acquire different functional properties.

EXPERIMENTAL PROCEDURES

Mice

NOD/SCID/IL-2Rγ-null mice transgenic for human SCF, IL3, and GM-CSF (NSG-SGM3) were bred at the Francis Crick Institute under specific pathogen-free conditions. Age- and sex-matched mice were used for all experiments. All experiments were performed in accordance with national and institutional guidelines for animal care and were approved by the Francis Crick Institute Animal Ethics Committee and by the United Kingdom Home Office.

Human Cells

Umbilical cord blood from healthy neonates was obtained from the Anthony Nolan Cell Therapy Centre under an agreement that includes ethical approval for laboratory research use. Mononuclear cells were obtained by density centrifugation using Ficoll-Paque (GE Healthcare) and ammonium chloride red cell lysis.

Flow Cytometry Analysis

Cells were stained in ice-cold PBS containing fetal calf serum (FCS, 2%) and EDTA (2 mM) using appropriate antibody-fluorophore conjugates. Prior to staining for DNGR-1, cells were pre-incubated on ice with mouse serum (Jackson ImmunoResearch Laboratories) and purified immunoglobulin G2a (IgG2a, BioLegend) to block Fc receptors. See the Supplemental Experimental Procedures for antibodies used. Multiparameter acquisition was performed on a Fortessa analyzer (BD Biosciences), and data were analyzed with FlowJo software (Tree Star). Prior to acquisition, cells were resuspended in PBS/FCS and 2%/EDTA (2 mM) solution with 1 μg/ml of DAPI to exclude dead cells.

Cell Sorting

Cells were sorted on a BD FACS Aria (BD Biosciences). For sorting of progenitors, mononuclear cells were isolated from umbilical cord blood, and lineage-negative cells were enriched using magnetic beads. Briefly, cells were incubated with Fc-block (BD Biosciences) for 10 min, stained with fluorescein isothiocyanate (FITC)-conjugated antibodies against lineage markers (Lin1-FITC) and washed before incubation with anti-FITC beads and enrichment on LD columns (both from Miltenyi Biotec). The flow-through fraction was stained with antibodies and sorted by FACS to achieve 99% purity. Dead cells were excluded using DAPI.

In Vitro Cultures

500–2,000 purified progenitors were cultured for 12 days in Iscove's Modified Dulbecco's Medium (IMDM) culture medium (Gibco) supplemented with β-mercaptoethanol and 10% heat-inactivated FCS at 37°C, together with the following cytokines (R&D Systems): human fms-like tyrosine kinase 3 ligand (hFLT3L) (100 ng/mL), human stem cell factor (hSCF) (20 ng/mL), human interleukin-4 (hIL-4) (20 ng/mL), and human granulocyte-macrophage colony-stimulating factor (hGM-CSF) (20 ng/mL). Half of the medium containing the cytokine cocktail was replaced every 3 days. In single-cell culture experiments, MS5 mouse fibroblast feeder cells were seeded in 96 well-culture plates (flat bottom) the day before to achieve 60%–70% confluence (3,000 MS5 cells/well). Single progenitors were then sorted directly onto the MS5 cell layer, and medium with cytokines was added subsequently.

In Vivo Transfer

Mice ages 8–12 weeks were sub-lethally irradiated (2 Gy) up to 24 hr before intravenous (i.v.) injection of 5,000–10,000 sorted CMPs, MLPs, or GMPs. The bone marrow of reconstituted mice was analyzed 2 weeks later.

Single-Cell qPCR

Single MLPs, CMPs, or GMPs were sorted directly into dry 96-well PCR plates and frozen at −80°C. See the Supplemental Experimental Procedures for cDNA production. qPCR for IRF8, MPO, and GAPDH was performed with TaqMan Universal PCR MasterMix (Applied Biosystems) and predesigned primers and probe mixes (TaqMan gene expression assays, Applied Biosystems). Measurements were performed using a sequence detection system (ABI Prism 7700, Applied Biosystems). The levels of mRNA for the specific gene being measured were divided by those for GAPDH measured in parallel (normalized expression).

Microarrays

$CD141^{+}DNGR\text{-}1^{+}$ cDC1 cells were sorted according to the gating strategy depicted in Figure 1B (upper panels) to achieve 99% purity. For each population, total RNA was extracted using an RNeasy Micro- or Minikit (QIAGEN). RNA was hybridized to the Affymetrix Human Gene 2.0 ST array according to the manufacturer's instructions. Each analysis was performed in triplicate using independently sorted cells from independent cultures. See the Supplemental Experimental Procedures for the microarray analysis.

Statistical Analysis

Statistical analyses were performed using GraphPad Prism as indicated in the figure legends. For the microarray analysis, differentially expressed genes were assessed using an empirical Bayes t test. The p values were adjusted using the Benjamini-Hochberg method. Microarrays data from a previous study used for comparative analysis are available under accession number GEO: GSE57671.

ACCESSION NUMBERS

The accession number for the microarray data reported in this paper is GEO: GSE98957.

SUPPLEMENTAL INFORMATION

Supplemental Information includes Supplemental Experimental Procedures and four figures and can be found with this article online at http://dx.doi.org/10.1016/j.celrep.2017.06.075.

AUTHOR CONTRIBUTIONS

Conceptualization, J.H., C.R.S., F.A.A., and D.B.; Methodology, J.H., C.R.S., F.A.A., and D.B.; Investigation, J.H., F.A.A., and A.G.v.d.V.; Formal Analysis, J.H., C.R.S., and P.C.; Writing – Original Draft, J.H. and C.R.S.; Writing – Review & Editing, J.H. and C.R.S.; Visualization, J.H.; Supervision, C.R.S.; Project Administration, J.H.; Funding Acquisition, C.R.S.

ACKNOWLEDGMENTS

We thank the Francis Crick Institute FACS laboratory for technical help. We are grateful to all members of the Immunobiology Laboratory and the Haematopoietic Stem Cell Laboratory for helpful discussions and comments. We thank Irene Sanjuan-Nandin from the Lymphocyte Biology Laboratory for advice and

Pierre Guermonprez for critical reading of the manuscript. This work was supported by the Francis Crick Institute, which receives core funding from Cancer Research UK (FC001136 and FC001045), the UK Medical Research Council (FC001136 and FC001045), and the Wellcome Trust (FC001136 and FC001045) and by ERC Advanced Investigator Grant AdG 268670 (to C.R.S.).

Received: February 18, 2017
Revised: May 22, 2017
Accepted: June 23, 2017
Published: July 18, 2017

REFERENCES

Akashi, K., Traver, D., Miyamoto, T., and Weissman, I.L. (2000). A clonogenic common myeloid progenitor that gives rise to all myeloid lineages. Nature *404*, 193–197.

Aliberti, J., Schulz, O., Pennington, D.J., Tsujimura, H., Reis e Sousa, C., Ozato, K., and Sher, A. (2003). Essential role for ICSBP in the in vivo development of murine CD8alpha + dendritic cells. Blood *101*, 305–310.

Balan, S., Ollion, V., Colletti, N., Chelbi, R., Montanana-Sanchis, F., Liu, H., Vu Manh, T.P., Sanchez, C., Savoret, J., Perrot, I., et al. (2014). Human XCR1+ dendritic cells derived in vitro from CD34+ progenitors closely resemble blood dendritic cells, including their adjuvant responsiveness, contrary to monocyte-derived dendritic cells. J. Immunol. *193*, 1622–1635.

Billerbeck, E., Barry, W.T., Mu, K., Dorner, M., Rice, C.M., and Ploss, A. (2011). Development of human CD4+FoxP3+ regulatory T cells in human stem cell factor-, granulocyte-macrophage colony-stimulating factor-, and interleukin-3-expressing NOD-SCID IL2Rγ(null) humanized mice. Blood *117*, 3076–3086.

Breton, G., Lee, J., Zhou, Y.J., Schreiber, J.J., Keler, T., Puhr, S., Anandasabapathy, N., Schlesinger, S., Caskey, M., Liu, K., and Nussenzweig, M.C. (2015). Circulating precursors of human CD1c+ and CD141+ dendritic cells. J. Exp. Med. *212*, 401–413.

Caux, C., Vanbervliet, B., Massacrier, C., Dezutter-Dambuyant, C., de Saint-Vis, B., Jacquet, C., Yoneda, K., Imamura, S., Schmitt, D., and Banchereau, J. (1996). CD34+ hematopoietic progenitors from human cord blood differentiate along two independent dendritic cell pathways in response to GM-CSF+TNF alpha. J. Exp. Med. *184*, 695–706.

Chicha, L., Jarrossay, D., and Manz, M.G. (2004). Clonal type I interferon-producing and dendritic cell precursors are contained in both human lymphoid and myeloid progenitor populations. J. Exp. Med. *200*, 1519–1524.

Crozat, K., Guiton, R., Contreras, V., Feuillet, V., Dutertre, C.A., Ventre, E., Vu Manh, T.P., Baranek, T., Storset, A.K., Marvel, J., et al. (2010). The XC chemokine receptor 1 is a conserved selective marker of mammalian cells homologous to mouse CD8alpha+ dendritic cells. J. Exp. Med. *207*, 1283–1292.

D'Amico, A., and Wu, L. (2003). The early progenitors of mouse dendritic cells and plasmacytoid predendritic cells are within the bone marrow hemopoietic precursors expressing Flt3. J. Exp. Med. *198*, 293–303.

Doulatov, S., Notta, F., Eppert, K., Nguyen, L.T., Ohashi, P.S., and Dick, J.E. (2010). Revised map of the human progenitor hierarchy shows the origin of macrophages and dendritic cells in early lymphoid development. Nat. Immunol. *11*, 585–593.

Fogg, D.K., Sibon, C., Miled, C., Jung, S., Aucouturier, P., Littman, D.R., Cumano, A., and Geissmann, F. (2006). A clonogenic bone marrow progenitor specific for macrophages and dendritic cells. Science *311*, 83–87.

Ginhoux, F., Liu, K., Helft, J., Bogunovic, M., Greter, M., Hashimoto, D., Price, J., Yin, N., Bromberg, J., Lira, S.A., et al. (2009). The origin and development of nonlymphoid tissue CD103+ DCs. J. Exp. Med. *206*, 3115–3130.

Grajales-Reyes, G.E., Iwata, A., Albring, J., Wu, X., Tussiwand, R., Kc, W., Kretzer, N.M., Briseño, C.G., Durai, V., Bagadia, P., et al. (2015). Batf3 maintains autoactivation of Irf8 for commitment of a CD8α(+) conventional DC clonogenic progenitor. Nat. Immunol. *16*, 708–717.

Greter, M., Helft, J., Chow, A., Hashimoto, D., Mortha, A., Agudo-Cantero, J., Bogunovic, M., Gautier, E.L., Miller, J., Leboeuf, M., et al. (2012). GM-CSF controls nonlymphoid tissue dendritic cell homeostasis but is dispensable for the differentiation of inflammatory dendritic cells. Immunity *36*, 1031–1046.

Guilliams, M., Ginhoux, F., Jakubzick, C., Naik, S.H., Onai, N., Schraml, B.U., Segura, E., Tussiwand, R., and Yona, S. (2014). Dendritic cells, monocytes and macrophages: a unified nomenclature based on ontogeny. Nat. Rev. Immunol. *14*, 571–578.

Hambleton, S., Salem, S., Bustamante, J., Bigley, V., Boisson-Dupuis, S., Azevedo, J., Fortin, A., Haniffa, M., Ceron-Gutierrez, L., Bacon, C.M., et al. (2011). IRF8 mutations and human dendritic-cell immunodeficiency. N. Engl. J. Med. *365*, 127–138.

Hildner, K., Edelson, B.T., Purtha, W.E., Diamond, M., Matsushita, H., Kohyama, M., Calderon, B., Schraml, B.U., Unanue, E.R., Diamond, M.S., et al. (2008). Batf3 deficiency reveals a critical role for CD8alpha+ dendritic cells in cytotoxic T cell immunity. Science *322*, 1097–1100.

Karsunky, H., Merad, M., Cozzio, A., Weissman, I.L., and Manz, M.G. (2003). Flt3 ligand regulates dendritic cell development from Flt3+ lymphoid and myeloid-committed progenitors to Flt3+ dendritic cells in vivo. J. Exp. Med. *198*, 305–313.

Kondo, M., Weissman, I.L., and Akashi, K. (1997). Identification of clonogenic common lymphoid progenitors in mouse bone marrow. Cell *91*, 661–672.

Lee, J., Breton, G., Oliveira, T.Y., Zhou, Y.J., Aljoufi, A., Puhr, S., Cameron, M.J., Sékaly, R.P., Nussenzweig, M.C., and Liu, K. (2015). Restricted dendritic cell and monocyte progenitors in human cord blood and bone marrow. J. Exp. Med. *212*, 385–399.

Liu, K., Victora, G.D., Schwickert, T.A., Guermonprez, P., Meredith, M.M., Yao, K., Chu, F.F., Randolph, G.J., Rudensky, A.Y., and Nussenzweig, M. (2009). In vivo analysis of dendritic cell development and homeostasis. Science *324*, 392–397.

Manz, M.G., Traver, D., Akashi, K., Merad, M., Miyamoto, T., Engleman, E.G., and Weissman, I.L. (2001). Dendritic cell development from common myeloid progenitors. Ann. N.Y. Acad. Sci. *938*, 167–173, discussion 173–174.

McGovern, N., Schlitzer, A., Gunawan, M., Jardine, L., Shin, A., Poyner, E., Green, K., Dickinson, R., Wang, X.N., Low, D., et al. (2014). Human dermal $CD14^{+}$ cells are a transient population of monocyte-derived macrophages. Immunity *41*, 465–477.

McKenna, H.J., Stocking, K.L., Miller, R.E., Brasel, K., De Smedt, T., Maraskovsky, E., Maliszewski, C.R., Lynch, D.H., Smith, J., Pulendran, B., et al. (2000). Mice lacking flt3 ligand have deficient hematopoiesis affecting hematopoietic progenitor cells, dendritic cells, and natural killer cells. Blood *95*, 3489–3497.

Merad, M., Sathe, P., Helft, J., Miller, J., and Mortha, A. (2013). The dendritic cell lineage: ontogeny and function of dendritic cells and their subsets in the steady state and the inflamed setting. Annu. Rev. Immunol. *31*, 563–604.

Murphy, T.L., Grajales-Reyes, G.E., Wu, X., Tussiwand, R., Briseño, C.G., Iwata, A., Kretzer, N.M., Durai, V., and Murphy, K.M. (2016). Transcriptional Control of Dendritic Cell Development. Annu. Rev. Immunol. *34*, 93–119.

Naik, S.H., Sathe, P., Park, H.Y., Metcalf, D., Proietto, A.I., Dakic, A., Carotta, S., O'Keeffe, M., Bahlo, M., Papenfuss, A., et al. (2007). Development of plasmacytoid and conventional dendritic cell subtypes from single precursor cells derived in vitro and in vivo. Nat. Immunol. *8*, 1217–1226.

Naik, S.H., Perié, L., Swart, E., Gerlach, C., van Rooij, N., de Boer, R.J., and Schumacher, T.N. (2013). Diverse and heritable lineage imprinting of early haematopoietic progenitors. Nature *496*, 229–232.

Onai, N., Obata-Onai, A., Schmid, M.A., Ohteki, T., Jarrossay, D., and Manz, M.G. (2007). Identification of clonogenic common Flt3+M-CSFR+ plasmacytoid and conventional dendritic cell progenitors in mouse bone marrow. Nat. Immunol. *8*, 1207–1216.

Persson, E.K., Uronen-Hansson, H., Semmrich, M., Rivollier, A., Hägerbrand, K., Marsal, J., Gudjonsson, S., Håkansson, U., Reizis, B., Kotarsky, K., and Agace, W.W. (2013). IRF4 transcription-factor-dependent CD103(+)CD11b(+) dendritic cells drive mucosal T helper 17 cell differentiation. Immunity *38*, 958–969.

Poulin, L.F., Salio, M., Griessinger, E., Anjos-Afonso, F., Craciun, L., Chen, J.L., Keller, A.M., Joffre, O., Zelenay, S., Nye, E., et al. (2010). Characterization of human DNGR-1+ BDCA3+ leukocytes as putative equivalents of mouse CD8alpha+ dendritic cells. J. Exp. Med. *207*, 1261–1271.

Poulin, L.F., Reyal, Y., Uronen-Hansson, H., Schraml, B.U., Sancho, D., Murphy, K.M., Håkansson, U.K., Moita, L.F., Agace, W.W., Bonnet, D., and Reis e Sousa, C. (2012). DNGR-1 is a specific and universal marker of mouse and human Batf3-dependent dendritic cells in lymphoid and nonlymphoid tissues. Blood *119*, 6052–6062.

Reya, T., Morrison, S.J., Clarke, M.F., and Weissman, I.L. (2001). Stem cells, cancer, and cancer stem cells. Nature *414*, 105–111.

Sathe, P., Vremec, D., Wu, L., Corcoran, L., and Shortman, K. (2013). Convergent differentiation: myeloid and lymphoid pathways to murine plasmacytoid dendritic cells. Blood *121*, 11–19.

Sathe, P., Metcalf, D., Vremec, D., Naik, S.H., Langdon, W.Y., Huntington, N.D., Wu, L., and Shortman, K. (2014). Lymphoid tissue and plasmacytoid dendritic cells and macrophages do not share a common macrophage-dendritic cell-restricted progenitor. Immunity *41*, 104–115.

Schlenner, S.M., Madan, V., Busch, K., Tietz, A., Läufle, C., Costa, C., Blum, C., Fehling, H.J., and Rodewald, H.R. (2010). Fate mapping reveals separate origins of T cells and myeloid lineages in the thymus. Immunity *32*, 426–436.

Schlitzer, A., McGovern, N., Teo, P., Zelante, T., Atarashi, K., Low, D., Ho, A.W., See, P., Shin, A., Wasan, P.S., et al. (2013). IRF4 transcription factor-dependent CD11b+ dendritic cells in human and mouse control mucosal IL-17 cytokine responses. Immunity *38*, 970–983.

See, P., Dutertre, C.A., Chen, J., Günther, P., McGovern, N., Irac, S.E., Gunawan, M., Beyer, M., Händler, K., Duan, K., et al. (2017). Mapping the human DC lineage through the integration of high-dimensional techniques. Science *356*, eaag3009.

Shortman, K., Sathe, P., Vremec, D., Naik, S., and O'Keeffe, M. (2013). Plasmacytoid dendritic cell development. Adv. Immunol. *120*, 105–126.

Steinman, R.M., Hawiger, D., Liu, K., Bonifaz, L., Bonnyay, D., Mahnke, K., Iyoda, T., Ravetch, J., Dhodapkar, M., Inaba, K., and Nussenzweig, M. (2003). Dendritic cell function in vivo during the steady state: a role in peripheral tolerance. Ann. N Y Acad. Sci. *987*, 15–25.

Traver, D., Akashi, K., Manz, M., Merad, M., Miyamoto, T., Engleman, E.G., and Weissman, I.L. (2000). Development of CD8alpha-positive dendritic cells from a common myeloid progenitor. Science *290*, 2152–2154.

Tussiwand, R., Everts, B., Grajales-Reyes, G.E., Kretzer, N.M., Iwata, A., Bagaitkar, J., Wu, X., Wong, R., Anderson, D.A., Murphy, T.L., et al. (2015). Klf4 expression in conventional dendritic cells is required for T helper 2 cell responses. Immunity *42*, 916–928.

Villani, A.C., Satija, R., Reynolds, G., Sarkizova, S., Shekhar, K., Fletcher, J., Griesbeck, M., Butler, A., Zheng, S., Lazo, S., et al. (2017). Single-cell RNA-seq reveals new types of human blood dendritic cells, monocytes, and progenitors. Science *356*, eaah4573.

Cell Reports, Volume *20*

Supplemental Information

Dendritic Cell Lineage Potential

in Human Early Hematopoietic Progenitors

Julie Helft, Fernando Anjos-Afonso, Annemarthe G. van der Veen, Probir Chakravarty, Dominique Bonnet, and Caetano Reis e Sousa

Figure S1

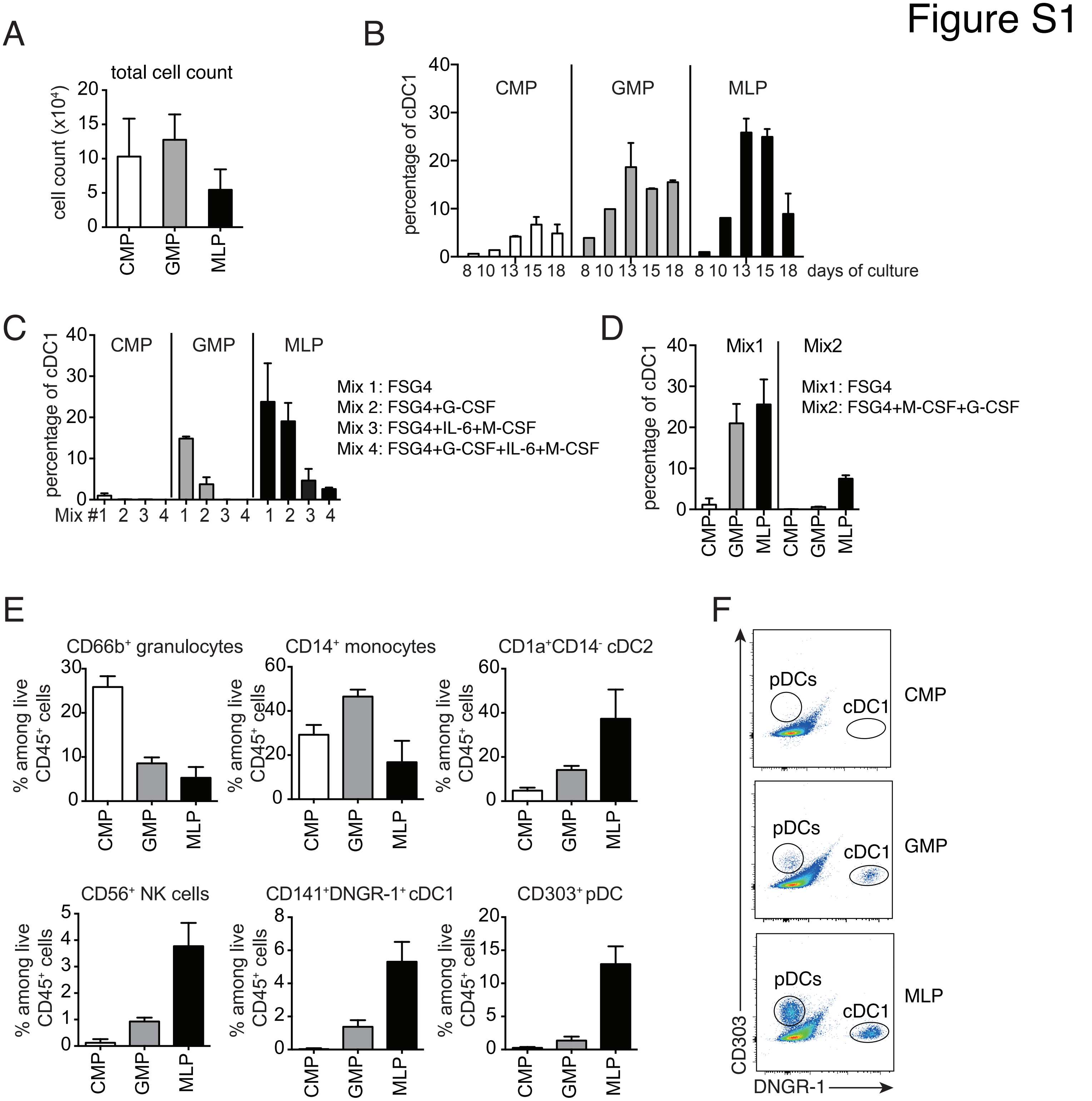

Figure S1. Expansion and cDC1 differentiation potential of CMPs, GMPs and MLPs in vitro. Related to Figure 1.
(A) The indicated progenitors were cultured for 12 days in vitro with FLT3-L, IL-4, SCF and GM-CSF (FSG4). Total cell count was determined by flow cytometry using counting beads. Data are average of 3 replicate wells from one experiment representative of at least 4 experiments. (B) CMPs, GMPs and MLPs were cultured for 8, 10, 13, 15 and 18 days in vitro with FSG4. Percentage of $CD141^+DNGR\text{-}1^+$ cDC1s was determined by flow cytometry. Data show one experiment representative of 2 experiments. (C-D) CMPs, GMPs and MLPs were cultured for 12 days with the indicated mixes of cytokines. $CD141^+DNGR\text{-}1^+$ cDC1 generation was assessed by flow cytometry. Data are average of 3 replicate wells from one experiment. (E-F) CMPs, GMPs and MLPs were cultured for 15 days with FLT3-L, SCF and GM-CSF (FSG) onto a layer of MS5 fibroblasts. (E) $CD141^+DNGR\text{-}1^+$ cDC1, $CD1c^+$ cDC2, $CD303^+$ pDCs, $CD56^+$ NK cells, $CD14^+$ monocytes and $CD66b^+$ granulocytes generation were assessed by flow cytometry. Data are average of 3 replicate wells from one experiment representative of 2 experiments. (F) $DNGR\text{-}1^+$ cDC1 and $CD303^+$ pDCs differentiation was assessed by flow cytometry. Dot plot show that $CD303^+$ pDCs do not express DNGR-1.

Figure S2

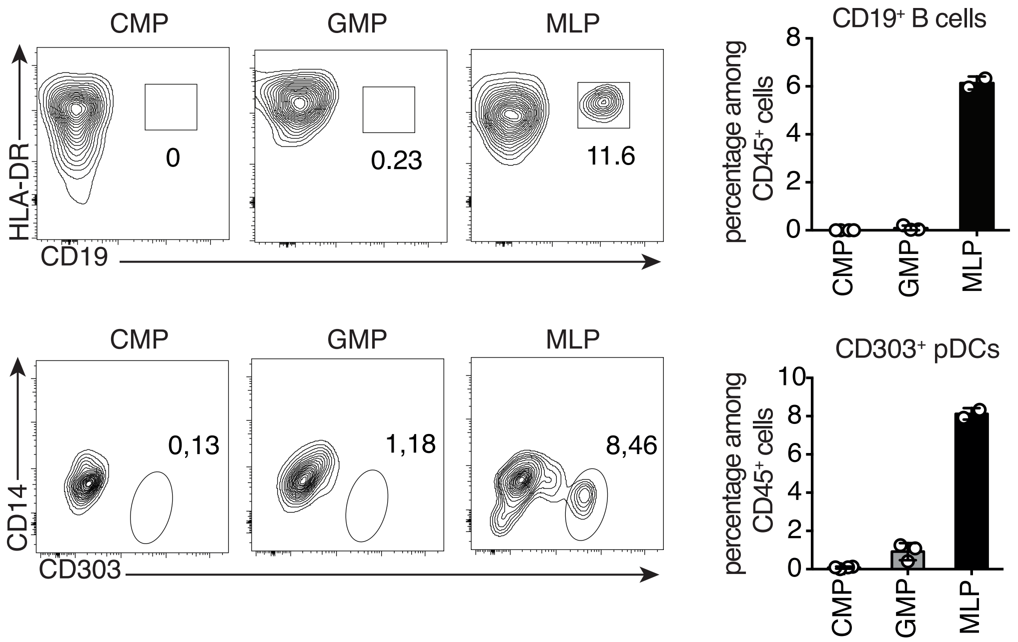

Figure S2. Plasmacytoid DC and B cell production in vivo. Related to Figure 2.
Irradiated NSG-SGM3 mice were injected intravenously with different purified human progenitors. Two weeks later, human B cell and plasmacytoid DC (pDC) presence in the bone marrow was quantitated by flow cytometry. Contour plots show the production of $CD19^+$ B cells and $CD303^+$ pDCs by the different progenitors. Graphs represent the percentage of each cell type among human $CD45^+$ cells engrafted in the bone marrow. Each dot represents an individual mouse, bars indicate the mean and error bars indicate the standard deviation.

Figure S3

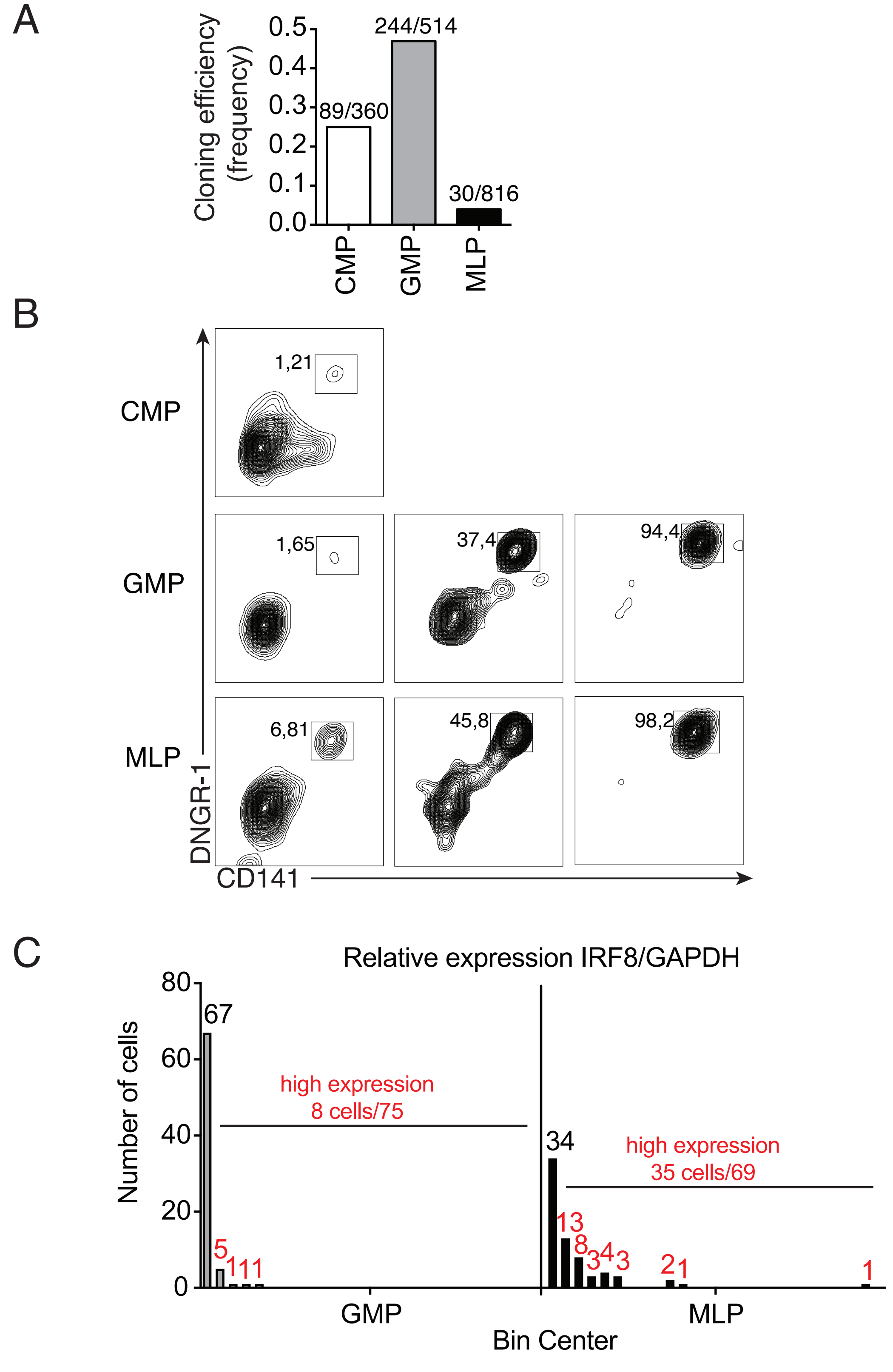

Figure S3. Single cell culture and single cell qPCR. Related to Figure 3.
(A) Cloning efficiency of single cell culture experiments from Figure 3A-C. Graph represents the frequency of successful clones among the total number of seeded wells.
(B) Examples of cDC1 generation in single cell cultures. Dot plots illustrate the % cDC1 that was detected in different wells seeded with CMPs, MLPs or GMPs.
(C) Single progenitors were sorted by flow cytometry into PCR plates (Figure 3D). IRF8 and GAPDH expression was measured for each well by RT-PCR. Bar graphs represent the number of single progenitors expressing different levels of IRF8 relative expression to GAPDH in the same cell. The actual number of cells for each level of expression is indicated on top of each bar. Data are a pool of two independent experiments.

Figure S4

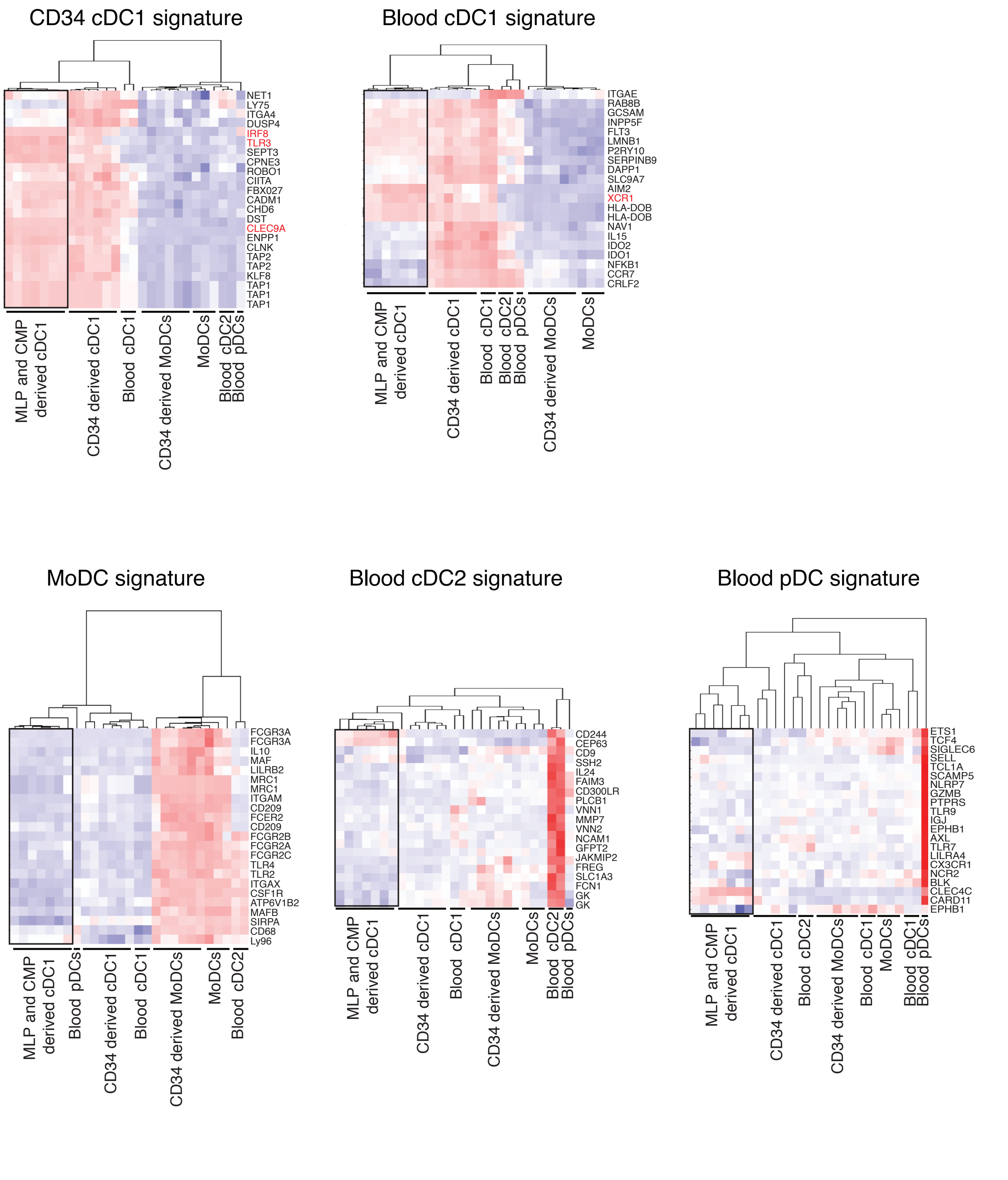

Figure S4. MLP- and CMP-derived cDC1 transcriptomic analysis. Related to Figure 4.
Heat maps of gene expression values comparing our MLP- and CMP-derived cDC1 populations with signatures from a published dataset of cord blood CD34+ cell-derived cDC1 and MoDC, as well as MoDC derived from isolated monocytes and primary cDC1, cDC2 and pDCs from peripheral blood (Balan et al., 2014). Individual replicates are shown.

SUPPLEMENTAL EXPERIMENTAL PROCEDURES

Flow cytometry analysis

Anti-HLA-DR (G46.6), anti-CD34 (8G12), anti-CD10 (HI10a), anti-CD19 (HIB19), and anti–Lineage-1 cocktail (CD3, CD14, CD16, CD19, CD20, and CD56) were purchased from BD. Anti-CD123 (6H6), anti-CD45RA (HI100), anti-CD38 (HIT2), anti-CD45 (2D1), anti-CD11c (3.9), anti-CD14 (61D3) were purchased from eBioscience. Anti-DNGR-1 (8F9) and purified isotype control were purchased from Biolegend. Anti-BDCA-1/CD1c (AD5-8E7) and anti-BDCA-3/CD141 (AD5-14H12) were purchased from Miltenyi Biotec. Isotype-matched irrelevant specificity control mAbs were used to control for unspecific staining.

Single cell qPCR

For cDNA production, cells were lysed in 7.2µl of a mix containing 0.51µl of NP40 10%, 0.37µl DTT, 0.15µl of rRNasin (Promega) and 1.03µl of random hexamer primers (Applied Biosystems), during 1 minute at 65°C. Then cDNA was synthesized from total RNA by adding 7µl of a second mix containing: 0.5µl of dNTP (Roche), 1µl DTT, 0.2µl rRNasin (Promega) and 0.3µl Superscript III (Invitrogen). Plate was incubated for 15 minutes at room temperature and then RT-PCR reaction was performed as follows: 5 minutes at 42°C, 10 minutes at 25°C, 55 minutes at 50°C and 5 minutes at 94°C.

Microarray analysis

Data were analysed using Bioconductor 2.13 (http://www.bioconductor.org) running on R 3.0.2 (available from www.R-project.org). Probeset expression measures were calculated using the Affymetrix package's Robust Multichip Average (RMA) default method. Differentially expressed genes were assessed between samples using an empirical Bayes t-test (limma package). P values were adjusted for multiple testing using the Benjamini-Hochberg method. Any probe sets that exhibited an adjusted P value of 0.05 were called differentially expressed. Two-dimensional hierarchical clustering of expression data was carried out using a 1 − Pearson correlation distance matrix and average linkage clustering. Genes were clustered using a Euclidean distance matrix. For heat map analysis, colors indicate the expression value relative to the mean expression value per gene in the dataset. Red indicates upregulation and blue indicates downregulation relative to mean value.
